# Assessment of IMPT versus VMAT plans using different uncertainty scenarios for prostate cancer

**DOI:** 10.1186/s13014-022-02126-y

**Published:** 2022-09-29

**Authors:** Michael P. Butkus, Nellie Brovold, Tejan Diwanji, Yihang Xu, Mariluz De Ornelas, Alan Dal Pra, Matt Abramowitz, Alan Pollack, Nesrin Dogan

**Affiliations:** 1 Department of Radiation Oncology, University of Miami Miller School of Medicine, 1475 NW 12th Ave, Miami, Florida 33136 USA; 2 Department of Radiation Oncology, Mayo Clinic, 200 First St. SW, Minnesota Rochester, 55905 USA; 3 Department of Radiation Oncology, Mid-Atlantic Permanente Medical Group, 1701 Twin Springs Rd, Maryland Halethrope, 21227 USA

**Keywords:** IMPT, Robust optimization, Prostate cancer

## Abstract

**Background:**

To assess the impact of systematic setup and range uncertainties for robustly optimized (RO) intensity modulated proton therapy (IMPT) and volumetric modulated arc therapy (VMAT) plans in patients with localized prostate cancer.

**Methods:**

Twenty-six localized prostate patients previously treated with VMAT (CTV to PTV expansion of 3-5 mm) were re-planned with RO-IMPT with 3 mm and 5 mm geometrical uncertainties coupled with 3% range uncertainties. Robust evaluations (RE) accounting for the geometrical uncertainties of 3 and 5 mm were evaluated for the IMPT and VMAT plans. Clinical target volume (CTV), anorectum, and bladder dose metrics were analyzed between the nominal plans and their uncertainty perturbations.

**Results:**

With geometric uncertainties of 5 mm and accounting for potential inter-fractional perturbations, RO-IMPT provided statistically significant (*p* < 0.05) sparing at intermediate doses (V_4000cGy_) to the anorectum and bladder and high dose sparring (V_8000cGy_) to the bladder compared to VMAT. Decreasing the RO and RE parameters to 3 mm improved IMPT sparing over VMAT at all OAR dose levels investigated while maintaining equivalent coverage to the CTV.

**Conclusions:**

For localized prostate treatments, if geometric uncertainties can be maintained at or below 3 mm, RO-IMPT provides clear dosimetric advantages in anorectum and bladder sparing compared to VMAT. This advantage remains even under uncertainty scenarios. As geometric uncertainties increase to 5 mm, RO-IMPT still provides dosimetric advantages, but to a smaller magnitude.

## Background

Intensity modulated proton therapy (IMPT) has the innate ability to modulate dose deposition in the beam direction through energy selection as well as radially by magnetic steering. By varying these parameters, a proton Bragg peak (known as a “spot”) can theoretically be placed in any 3-dimensional coordinate. Adjustments of these parameters, as well as the intrinsic inverse depth-dose profile and rapid dose fall-off of proton pencil beams allows IMPT to provide conformal plans while minimizing dose to healthy tissues [1, 2] As a result, for a multitude of treatment sites, IMPT has been shown to be better able to spare healthy organs at risk (OARs) while maintaining clinical target volume (CTV) dose coverage compared to other advanced treatment modalities, such as photon-based volumetric modulated arc therapy (VMAT) [3–6].

The greater sparing potential of IMPT is often at the cost of greater uncertainties in treatment planning and delivery [7–9]. Small misalignments of a patient or changes in internal anatomy degrade the quality of proton treatment plans more readily than in photon plans. Additionally, IMPT must account for uncertainties in the conversion of CT Hounsfield Units (HU) into proton stopping power ratios (SPR) [10]. This uncertainty propagates itself as uncertainty in the exact depth of the Bragg peak and is specific to ion therapy. To mitigate the impact of these uncertainties, the standard of care for IMPT is to robustly optimize (RO) to a CTV accounting for rigid body translations (usually ± 3–5 mm in cardinal directions) and for adjustments in the HU to SPR calibration curve (usually ± 3–5%). For IMPT, RO approaches have been shown to produce treatment plans less susceptible to setup and range uncertainties than optimization to a conventional planning target volume (PTV) that is geometrically expanded from a CTV [11–13]. Despite these differences, the same logic of selecting margins for photon-based PTV expansions apply to the selection of input parameters for RO-IMPT [3]. These parameters must be large enough to account for adequate coverage of a CTV in realistic set-up and range uncertainty scenarios while also minimizing its size to prevent unnecessary dose to OARs. When selecting a patient for either photon or proton therapy, knowledge of the potential geometric uncertainty is important. Small geometric uncertainties favor proton therapy treatment, in which the rapid dose falloff of the Bragg Peak can be fully exploited. While larger geometric uncertainties become less advantageous for proton treatments due to the increased sensitivity of this treatment to deviations in setup and range. Therefore, patient selection for proton therapy necessitates knowledge of the threshold of geometric uncertainties above which the modality is no longer advantageous over VMAT; and if these uncertainties are clinically achievable [6, 14, 15].

Further uncertainties in IMPT are derived from the method in which spot intensity and location are optimized to create a desirable dose distribution. Single field optimization (SFO) techniques optimize spot intensities with the goal of each individual field being able to independently meet optimization objectives, ignorant of the dose that could be contributed by other fields [16]. Contrarily, multifield optimization (MFO) techniques optimize spot intensities from multiple fields collectively with the goal of the overall plan meeting optimization objectives [17]. MFO plans offer more flexibility to create ideal dose distributions, especially for complicated anatomies [18]. However, compared to SFO, MFO spot intensities can be highly non-uniform and individual fields do not necessarily provide dose coverage to an entire target volume [19]. This results in MFO dose distributions being more sensitive to the uncertainties of proton therapy. Due to this sensitivity, MFO plans are generally not the treatment of choice for simple anatomies in which MFO does not provide obvious dosimetric advantages, such as the simple treatment anatomy of many localized prostate plans. However, if MFO plans can provide satisfactory planning robustness as to not be affected by these uncertainties, the added degrees of freedom of MFO planning may allow them to be better able be to spare OARs as the individual treatment fields can be more customizable than SFO. In this work we will not be comparing SFO to MFO robustness, but rather MFO to VMAT robustness under the assumption that if MFO plans can provide robustness on the level of VMAT then MFO itself can be considered when triaging a patient between VMAT and IMPT.

Previous studies have compared nominal IMPT plans to nominal VMAT plans showing a superior homogeneity index and OAR dose sparing. Ong et al. evaluated the robustness on the PTV-based, SFO, IMPT plans created for whole pelvic irradiation in prostate cancer and compared it to the nominal photon (VMAT) plan [20]. Goddard et al.compared the robust evaluations of VMAT and RO-IMPT treatments plans for 10 hypofractionated prostate SBRT patients, accounting for 2 mm setup uncertainty coupled with 1 or 3% range uncertainty [21]. They showed robustness of the VMAT plans outperformed IMPT plans in terms of target coverage and OAR sparing, despite the nominal comparability between VMAT and IMPT in terms of CTV coverage, conformity, and OAR sparing. For conventional fractionation, however, the use of margins greater than 2 mm and range uncertainties of 1% are more appropriate and therefore yield different robust evaluation results. Additionally, Pugh et al. evaluated the dosimetry of MFO-IMPT using robustness analysis of simulated rotational and translational alignment errors for ten localized prostate cancer patients [17] and showed that the rotational errors caused minimal dose perturbations in bladder and rectum. Although translational errors resulted in much larger dose perturbations in bladder and rectum, the target coverage was acceptable up to 5 mm translational errors.

A more recent work by Whitaker et al. showed significant sparing with IMPT as compared to VMAT for pelvic node irradiation of prostate cancer [15]. The study results showed adequate CTV coverage for both VMAT and IMPT although mean doses to the rectum, bladder, large and small bowel were lower using IMPT [15]. To our knowledge comparison of robust evaluations between multifield robustly optimized (MFO-RO) and VMAT for conventionally fractionated treatment of prostate cancer has not been previously investigated. The goal of this work was to determine the setup uncertainty required for MFO-RO proton therapy to be dosimetrically advantageous over VMAT for localized prostate cancer patients.

## Materials and methods

### Patient cohort

Twenty-six patients with localized prostate cancer, previously treated with VMAT were selected for this study. All patients had what could be considered “normal” anatomy and there were no implanted hip prostheses, intrarectal spacers, or rectal balloons. The prostate gland of each patient was implanted with 4 fiducial markers. A planning CT (pCT) with 1.5 mm slices thickness was acquired with the patient supine and with a full bladder. External immobilization and patient support were purposely kept to a minimum as all patients receive daily CBCTs on a 6D couch for alignment and patients had internal fiducials for matching. Support devices only included a hand ring to keep the patients arms on their chest, a foam mat for back comfort, and lower leg supports to maintain straightness of the legs. Patients would be adjusted to ensure straightness of the pelvis and symmetry of the femoral heads before acquisition of the pCT and then marked laterally and anteriorly to aid in initial treatment setup. For contouring assistance, pCTs were fused (MIM Software Inc., Cleveland, OH, ver. 6.7) with T2-Fast Spin Echo (T2-FSE), T2-Fast Spin Echo Fat Saturated (T2-FSE-FS), Apparent Diffusion Coefficient (ADC), T2*, and early and late stage T1-based Dynamic Contrast Enhancement (DCE) subtraction MRI sequences. For all patients, the CTV consisted of the entire prostate gland and proximal seminal vesicles. Target volumes prescription doses varied between patients with 13 of the 26 receiving a prescribed dose of 7600 cGy and the other 13 receiving a prescribed dose of 8000 cGy. All treatments were delivered in 200 cGy fractions.

### VMAT planning

All VMAT plans were clinically treated plans consisting of 2–4 arcs. PTVs were created as a 3 mm expansion from the CTV posteriorly and 5 mm in all other dimensions. The asymmetric expansions are clinically used to allow for greater sparing of the rectum. Clinically, all plans achieved 100% prescription dose coverage to at least 95% of the PTV. However, for comparison purposes in this study, normalization was set so that full prescription coverage was achieved to exactly 95% of the PTV volume. All plans had the same initial OAR sparing goals, which are given in Table 1. The VMAT plans were generated using Eclipse treatment planning system (TPS) and utilized Acuros XB dose calculation algorithm with 2 mm grid size (Varian Medical Systems, Palo Alto, California, ver. 15.6).

**Table 1:** Clinical dose constraints for plan evaluations and initial optimization objectives for VMAT and IMPT plans

Structure	Dose	Volume constraint
CTV	95% D_RX_	≥ 95%
	Max	≤ 115% D_RX_
Anorectum	4000 cGy	≤ 35%
	6500 cGy	≤ 17%
	8000 cGy	≤ 10%
Bladder	4000 cGy	≤ 50%
	6500 cGy	≤ 25%
	8000 cGy	≤ 10%

To analyze dosimetric variations from setup uncertainties, each VMAT plan was robustly evaluated (RE) by applying isocenter shifts of ± 3 mm or ± 5 mm in the anterior/posterior, left/right, and superior/inferior directions. For each shift, the fluence from its nominal VMAT plan was then used to calculate the altered dose deposition resulting from the shift. Overall, 13 dose distributions were created for each VMAT patient. A single dose distribution from the nominal plan (VMAT_orig_), 6 dose distributions from the ± 3 mm shifts in the cardinal directions (VMAT_3mm_), and 6 dose distributions from the analogous ± 5 mm shifts (VMAT_5mm_).

### IMPT planning

All IMPT plans consisted of two parallel-opposed lateral fields. Field specific targets were created around the CTV to restrict potential spot placement. Laterally, these targets restricted spots to within 10 mm of the CTV. In the beam-direction spots were restricted to within 10 mm plus 3% of the beam range. For optimization, MFO was implemented using Nonlinear Universal Proton Optimizer (Eclipse, Varian Medical Systems, Palo Alto, California, NUPO 15.6) and dose calculations were performed using Proton Convolution Superposition (Eclipse, Varian Medical Systems, Palo Alto, California, PCS 15.6) algorithms. A 2 mm calculation grid size was used for both algorithms. All IMPT dose calculations assumed a constant relative biological effectiveness (RBE) of 1.1. Spot spacing was beam energy specific and was set as one sigma (0.425 FWHM) of the beams Gaussian profile in air, at isocenter (3.5–4.8 mm for representative energies corresponding to Bragg peak tissue depths of 10–30 cm). Energy layers were uniformly spaced at 3.5 MeV intervals. RO was only applied to the CTV, with the goal of ensuring 95% of the CTV volume receiving 100% of the prescription dose under all uncertainty perturbations. All IMPT plans were created using a previously validated [22] Proton RapidPlan^™^ model (Eclipse ver. 16.0) with the same OAR dose sparing goals as the VMAT plans. After optimization, all nominal plans were normalized to full prescription dose to 100% of the CTV. For each patient, two separate nominal IMPT plans were generated: one with RO uncertainty perturbations of ± 3 mm in the anterior/posterior, left/right, and superior/inferior directions coupled with ± 3% range uncertainty (IMPT_RO3_) and another with the same range uncertainty but with ± 5 mm geometric uncertainty perturbations (IMPT_RO5_).

For each patient, in addition to the two nominal plan dose distributions, supplemental dose distributions were created by projecting the same proton spot pattern on isocenter shifts and range uncertainties that were equivalent to the RO uncertainty perturbations. This resulted in 12 supplemental dose distributions for ± 3 mm and ± 3% range uncertainties (IMPT_3%/3 mm_) and 12 supplemental dose distributions for ± 5 mm and ± 3% range uncertainties (IMPT_3%/5 mm_) giving each patient 26 total IMPT dose distribution scenarios.

### Normalization of VMAT and IMPT plans

VMAT plans were normalized so that 95% of the PTV received full prescription dose. However, IMPT plans were normalized so that 100% of the CTV received full prescription coverage in the nominal plan. Additionally, RO objectives were set to try to ensure 95% of the CTV received full prescription under uncertainty scenarios. This creates a situation in which the normalization between the comparison plans is not equivalent. However, we used this method as it represents our institutions planning goals for both VMAT and IMPT plans. In VMAT planning the usage of a PTV is to ensure that the CTV receives adequate coverage, even with uncertainties. In RO-IMPT planning, the RO objectives serve the same goal of ensuring CTV coverage, even with uncertainties. By setting the RO objectives of the CTV in IMPT planning to the same prescription objective of the PTV in VMAT planning the intent of the plans is equivalent, that is to ensure 95% of the target volume receives full prescription dose, even with uncertainties.

### Analysis of nominal plan dose distributions

Comparison of the VMAT nominal plans against the IMPT nominal plans is important to identify which modality can deliver a preferential treatment in an optimal scenario without uncertainties. This gives a baseline dose-distribution against which the effect of uncertainties can be analyzed. For each patient, nominal plans were compared according to target dose-volume (DV) and OAR DV metrics. Target DV metrics were based on relative target hotspots (D_max_), conformity index (CI) [23], volumetric coverage of the 95% prescription isodose (V_95_), and the 98% to 2% homogeneity index (HI) [24], defined as:

1 $$HI = \frac{{D_{2} - D_{98} }}{{D_{rx} }}$$

where ${D}_{2}$  is the minimum dose to 2% of the target volume, ${D}_{98}$ is the minimum dose to 98% of the target volume, and  ${D}_{rx}$ is the prescription dose. In this formalization, 2% is used to represent a practical maximum dose while ${D}_{98}$ represents a practical minimum dose. Smaller HI values indicate greater dose homogeneity within the target volume. The CI was defined as the ratio of the 100% isodose volume to the volume of the CTV. CI values below 1 indicate underdosing of a CTV, while values above 1 indicate excessive dose being deposited in healthy tissues near the CTV. OAR DV metrics were evaluated against the original DV optimization objectives for the anorectum and bladder (volume of organ receiving ≥ 4000 cGy (V_4000cGy_), 6500 cGy (V_6500cGy_), and 8000 cGy (V_8000cGy_), respectively).

### Analysis of uncertainty dose distributions

Uncertainty analysis was completed by analyzing the potential probability distribution and ranges of the same metrics analyzed on the nominal plans, but for each uncertainty scenario. For all uncertainty scenarios, dose was accumulated as if the uncertainty was a systematic error for all treatment fractions.

### Statistical analysis

Statistical differences between different group cohorts (nominal dose evaluations: VMAT_orig_, IMPT_RO3_, IMPT_RO5_. Uncertainty evaluations: VMAT_3mm_, VMAT_5mm_, IMPT_3%/3 mm_, IMPT_3%/5 mm_) was assessed using the two-way Wilcoxon rank sum test. Statistically significant differences were defined as having a *p*-value < 0.05. JMP Pro (SAS Institute, Cary, North Carolina) software was used for all statistical analyses.

## Results

### Nominal plan evaluations

**Fig. 1:**
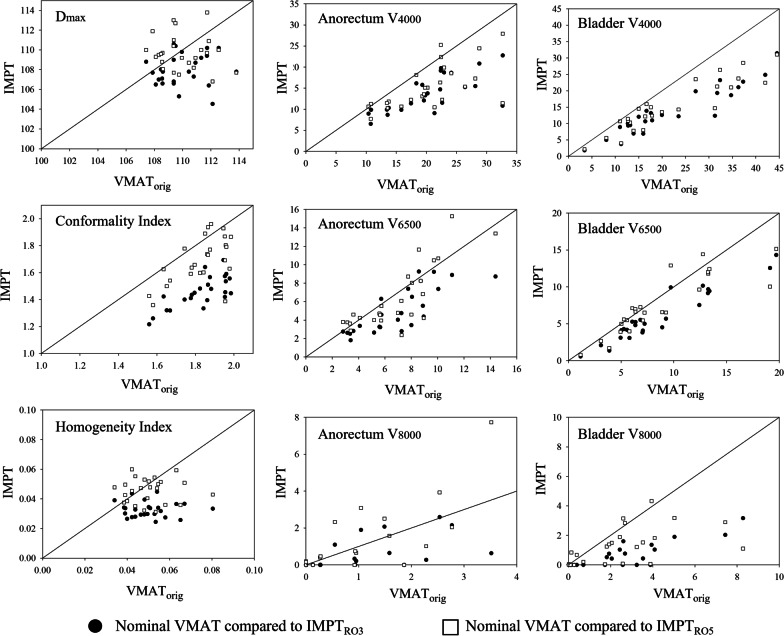
Patient-specific comparison of VMAT_orig_ to IMPT_RO3_ and IMPT_RO5_ nominal cohorts. In all panels, filled circles represent comparisons between patients VMAT_orig_ and IMPT_RO3_ dose-distributions. Hollow squares represent comparisons between VMAT_orig_ and IMPT_RO5_ dose-distributions. All marks below the diagonal identity line indicate that IMPT plans were preferable to VMAT for the specific dose metric in each panel. Left column panels displays CTV statistics. From top to bottom, D_max_, CI, and HI metrics are shown in this column. Middle column displays anorectum dose metrics and right column displays bladder dose metrics. For both columns, from top to bottom, V_4000cGy_, V_6500cGy_, and V_8000cGy_ metrics are shown for each organ.

Figure 1 shows comparisons between the nominal dose metrics for the VMAT_orig_ cohort and the IMPT_RO_ cohort for each of the twenty-six patients. Additionally, Table 2 gives the mean, standard deviations, and range of dose metrics for all patients in the 3 different nominal cohorts.

**Table 2:** CTV D_max_, HI, CI, V95 and anorectum/bladder V_4000cGy_, V_6500cGy_, and V_8000cGy_ dose statistics for all cohorts analyzed

	Nominal Plans			Uncertainty Plans			
Cohort	VMAT_orig_	IMPT_RO3_	IMPT_RO5_	VMAT_3mm_	IMPT_3%/3 mm_	VMAT_5__mm_	IMPT_3%/5 mm_
*CTV D*_*max*_							
Mean	110.0 ± 1.7	**108.0 ± 1.6**	109.7 ± 1.8	110.0 ± 1.7	**106.8 ± 1.1**	110.0 ± 1.8	***108.7* ± *1.9***
Range	107.4–113.8	104.5–110.6	106.8–113.8	106.7–114.5	104.2–110.0	106.7–114.4	104.0–114.6
Failure rate of uncertainty plans (%)				0.0	0.0	0.0	0.0
*CTV homogeneity index (HI)*							
Mean	0.05 ± 0.01	**0.03 ± 0.01**	0.05 ± 0.01	**0.05 ± 0.01**	0.06 ± 0.02	*0.07* ± *0.03*	0.09 ± 0.04
Range	0.03–0.08	0.02–0.04	0.03–0.06	0.03–0.10	0.01–0.10	0.04–0.25	0.04–0.26
*CTV conformity index (CI)*							
Mean	1.8 ± 0.1	**1.5 ± 0.1**	**1.7 ± 0.2**	1.8 ± 0.1	**1.2 ± 0.1**	1.8 ± 0.1	**1.5 ± 0.2**
Range	1.6–2.0	1.2–1.7	1.4–2.0	1.6–2.0	0.8–1.6	1.6–2.0	1.0–2.0
*CTV V95%*							
Mean	99.9 ± 0.1	100.0 ± 0.0	100.0 ± 0.0	**99.9 ± 0.2**	99.6 ± 0.4	***99.7* ± *0.7***	98.8 ± 1.2
Range	99.7–100.0	100.0	100.0	98.3–100.0	97.5–100.0	94.5–100	95.4–100
Failure rate of uncertainty plans (%)				0.0	0.0	0.0	0.0
*Anorectum (V*_*4000cGy*_*)*							
Mean	20.4 ± 6.3	**13.8 ± 4.4**	**15.3 ± 5.3**	20.7 ± 8.2	**14.0 ± 6.2**	21.2 ± 10.2	**15.8 ± 9.0**
Range	10.4–32.7	6.6–22.8	7.7–27.9	5.00–45.7	3.0–32.0	2.1–53.7	1.7–42.5
Failure rate of uncertainty plans (%)				4.6	0.0	8.7	3.1
*Anorectum (V*_*6500cGy*_*)*							
Mean	7.0 ± 2.9	5.1 ± 2.5	6.4 ± 3.5	7.4 ± 4.9	**5.5 ± 4.0**	8.1 ± 6.5	7.4 ± 6.4
Range	2.8–14.4	1.8–9.3	2.4–15.3	0.1–22.8	0.1–17.0	0.0–28.9	0.0–27.1
Failure rate of uncertainty plans (%)				5.1	0.3	10.9	8.6
*Anorectum (V*_*8000cGy*_*)*							
Mean	0.9 ± 1.1	0.5 ± 0.8	1.1 ± 1.8	1.4 ± 2.3	**0.8 ± 1.5**	1.8 ± 3.0	1.9 ± 3.4
Range	0.0–3.5	0.0–2.6	0.0–7.7	0.0–12.3	0.0–7.1	0.0–12.4	0.0–17.7
Failure rate of uncertainty plans (%)				0.6	0	3.0	5.8
*Bladder (V*_*4000cGy*_*)*							
Mean	21.9 ± 11.4	**13.7 ± 7.3**	**15.3 ± 7.7**	21.9 ± 11.9	**13.5 ± 7.8**	21.9 ± 12.8	***15.1* ± *9.3***
Range	3.4–44.5	1.8–31.5	2.2–31.1	2.3–53.4	1.0–42.0	1.7–60.9	0.9–46.0
Failure rate of uncertainty plans (%)				1.1	0.0	1.7	0.0
*Bladder (V*_*6500cGy*_*)*							
Mean	8.5 ± 4.7	**6.0 ± 3.5**	7.4 ± 3.9	8.6 ± 5.4	**5.9 ± 4.4**	8.7 ± 6.5	*7.6* ± *5.9*
Range	1.2–19.7	0.6–14.3	0.8–15.2	0.6–28.4	0.2–25.1	0.3–34.7	0.1–30.7
Failure rate of uncertainty plans (%)				1.1	0.3	2.3	0.9
*Bladder (V*_*8000cGy*_*)*							
Mean	2.5 ± 2.3	**0.6 ± 0.9**	**1.3 ± 1.3**	2.7 ± 2.9	**0.6 ± 1.2**	2.9 ± 3.6	***1.4* ± *2.1***
Range	0.0–8.3	0.0–3.2	0.0–4.3	0.0–15.6	0.0–9.39	0.0–20.9	0.0–11.7
Failure rate of uncertainty plans (%)				2.4	0.0	4.8	0.6

Left and right sided columns give mean, standard deviations, minimum, and maximum doses statistics for all nominal and uncertainty cohorts, respectively. For each uncertainty cohort, the percentage of dose distributions that did not reach each clinical dose objective is additionally given. **Bold** and *Italicized* values indicate statistically significant differences between IMPT and VMAT cohorts with equivalent and non-equivalent geometric uncertainties, respectively. Underlined values indicate that the within similar modalities, one cohort significantly outperformed the other with different geometric uncertainties

Due to normalization, all nominal IMPT plans achieved acceptable volumetric CTV coverage (V_95%_ = 100%). Of the 26 nominal VMAT plans, one achieved slightly less than 95% CTV coverage even when the PTV was normalized to V_95%_ = 100%. The IMPT cohort with 3 mm geometric uncertainties had statistically significant lower maximum dose, HI, and CI than the VMAT and IMPT_3%/5 mm_ cohorts. The IMPT_3%/5 mm_ cohort had statistically significant lower conformity index than VMAT but were nearly equivalent to VMAT for maximum dose and HI.

In terms of OAR sparing, the greatest difference between IMPT and VMAT was observed in bladder doses. For the bladder, IMPT_RO3_ had statistically smaller irradiated volumes than VMAT for the three analyzed dose levels (V_4000cGy_, V_6500cGy_ and V_8000cGy_) and was significantly lower than the IMPT_RO5_ cohort at the V_8000cGy_ threshold. IMPT_RO5_ irradiated volumes were statistically less than VMAT at the V_4000cGy_ and V_8000cGy_ thresholds. The V_6500cGy_ volume of IMPT_RO5_ had lower numerical value than VMAT, however, the difference was not statistically significant.

The anorectum was better spared with IMPT as well. Mean IMPT irradiated volumes were lower than VMAT at all dose levels except for V_8000cGy_ for the IMPT_RO5_ cohort, but only reached statistical significance for V_4000cGy_. At this threshold, differences between IMPT_RO3_ and IMPT_RO5_ were not statistically significant.

### Evaluation of uncertainty scenarios

**Fig. 2:**
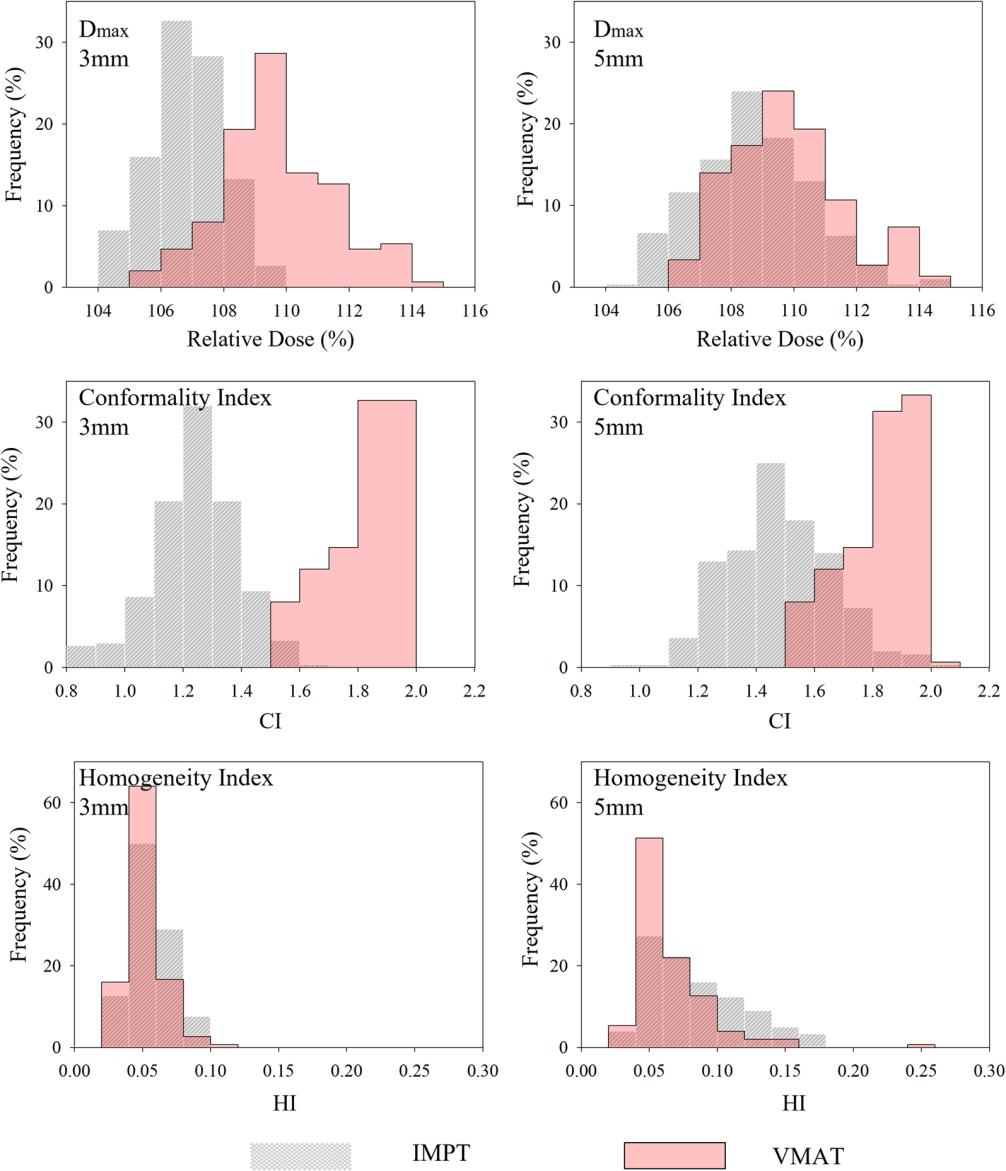
Relative frequency distribution of CTV dose metrics resulting from all uncertainty analysis. Grey hatched distribution is for IMPT dose metrics and red-filled distribution is for VMAT dose metrics. Left column shows comparison between dissimilar modalities with 3 mm geometric uncertainties and right column shows comparison between dissimilar modalities with 5 mm geometric uncertainties

**Fig. 3:**
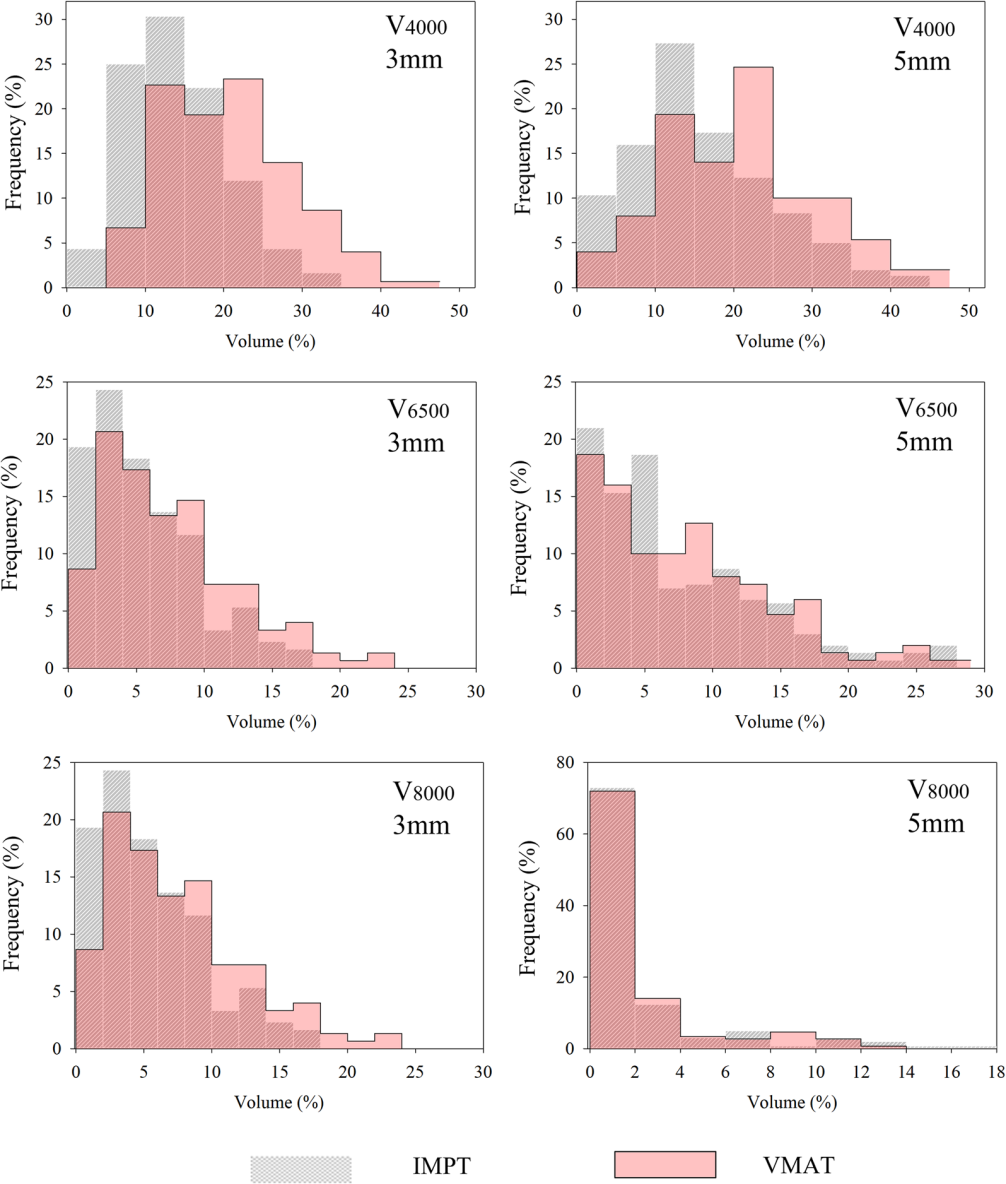
Relative frequency distribution of anorectum dose metrics resulting from all uncertainty analysis. Grey hatched distribution is for IMPT dose metrics and red-filled distribution is for VMAT dose metrics. Left column shows comparison between dissimilar modalities with 3 mm geometric uncertainties and right column shows comparison between dissimilar modalities with 5 mm geometric uncertainties

**Fig. 4:**
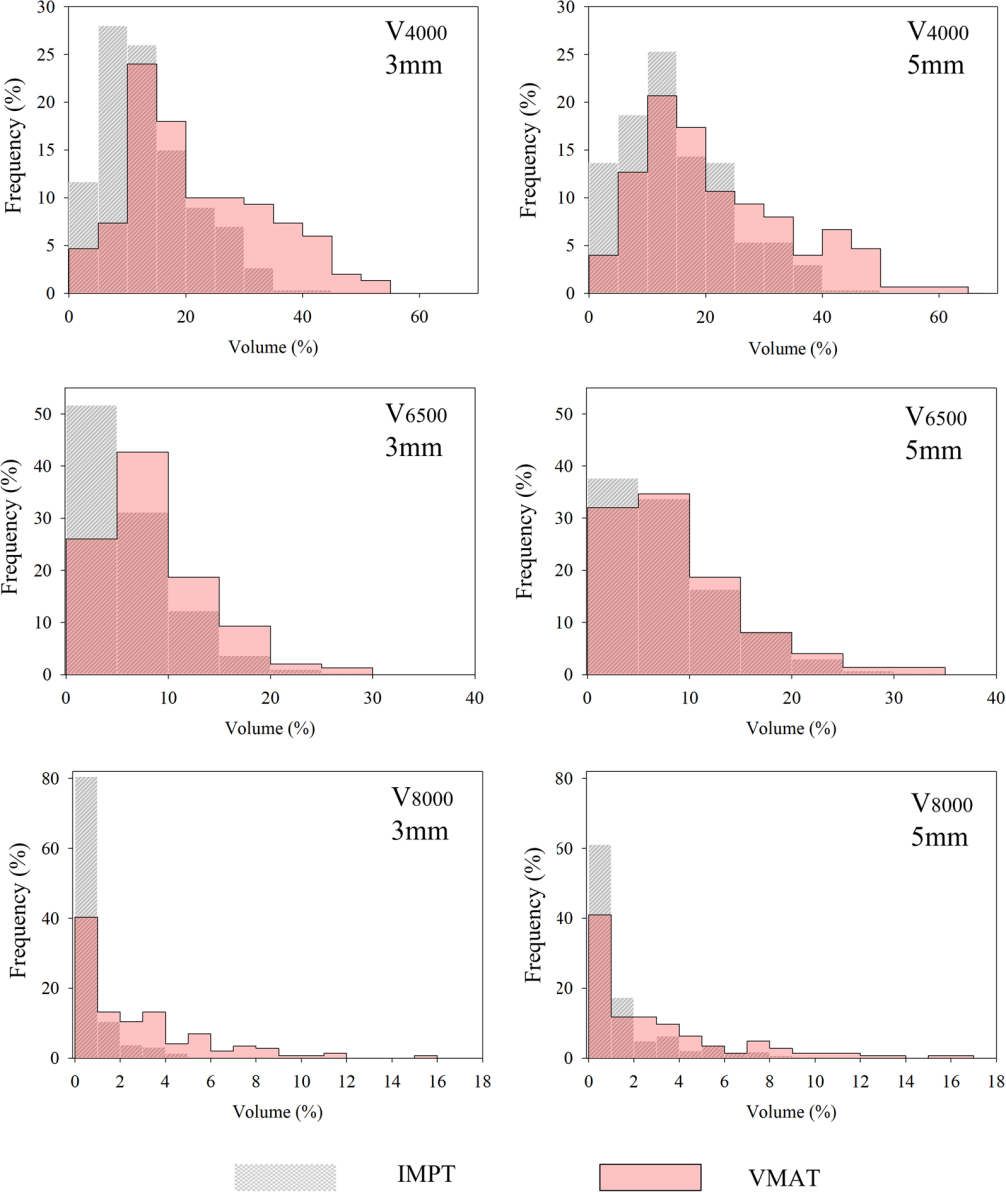
Relative frequency distribution of bladder dose metrics resulting from all uncertainty analysis. Grey hatched distribution is for IMPT dose metrics and red-filled distribution is for VMAT dose metrics. Left column shows comparison between dissimilar modalities with 3 mm geometric uncertainties and right column shows comparison between dissimilar modalities with 5 mm geometric uncertainties

Figures 2, 3, 4 show relative frequency plots of analyzed dose metrics for the 156 VMAT uncertainty scenarios superimposed on the 312 IMPT uncertainty scenarios for plans with analogous geometric uncertainties (VMAT_3mm_ vs. IMPT_3%/3 mm_ and VMAT_5mm_ vs. IMPT_3%/5 mm_). Additionally, Table 2 shows the mean, standard deviations, and range of dose metrics for each uncertainty cohort.

CTV coverage of V95% < 95% was maintained in all IMPT uncertainty scenarios and 155 of the 156 VMAT uncertainty scenarios indicating that CTV coverage is acceptable regardless of treatment modality. In the CTV, IMPT cohorts had significantly lower maximum doses and CIs than their analogous VMAT cohort. IMPT_3%/5 mm_ uncertainty plans even had significantly lower values of these metrics than the VMAT_3mm_ uncertainty cohort. The HI for all uncertainty cohorts were statistically distinguishable from one another with VMAT_3mm_ having the lowest HI, followed by IMPT_3%/3 mm_, VMAT_5mm_, and IMPT_3%/5 mm_, respectively. Statistically, VMAT uncertainty plans provided better CTV coverage at the V_95_ threshold, but absolute differences were minimal with all IMPT uncertainties maintaining V_95_ greater than 95%.

At the V_4000cGy_ threshold of the anorectum, both IMPT cohorts had significantly lower irradiated volumes than VMAT but were not indistinguishable from one another. At the higher dose thresholds, IMPT_3%/3 mm_ showed statistically better sparing of the anorectum than all other cohorts. In the bladder, like the nominal doses, IMPT plans statistically outperformed their VMAT counterparts except for V_6500cGy_ with 5 mm uncertainty. At the highest two dose thresholds, IMPT_3%/3 mm_ plans showed statistically better sparing than IMPT with larger geometric uncertainties.

## Discussion

Accounting for geometric uncertainties has a profound impact on the resultant dose distributions of an IMPT plan. Greater potential dose deviations from uncertainties with IMPT compared to VMAT warrants higher fidelity investigation and dose evaluation under different uncertainty scenarios. For many treatment sites the assessment of which treatment modality is most beneficial to a patient depends on the magnitude of the expected uncertainties. In this work the impact of IMPT planning for, and subsequently evaluating, localized prostate cancer treatment plans with 3- and 5-mm geometric uncertainties coupled with 3% range uncertainties was investigated and compared to clinical VMAT planning.

Minimal differences in CTV coverage was observed between the different cohorts. All nominal and uncertainty scenarios achieved clinical standards for adequacy of target coverage indicating that the ability to accurately deliver dose to the primary target volume is not a limiting factor in localized prostate therapies with VMAT or IMPT. IMPT plans were able to reach prescription dose levels while minimizing target hotspots compared to VMAT. This fact created a situation in which the nominal homogeneity of the CTV from IMPT was better than that of VMAT. However, when uncertainties were considered, the homogeneity of VMAT plans surpassed IMPT. This is due to the broader dose cloud created around the CTV for VMAT plans. For example, for a representative patient, the 95% isodose volume around a 61.7 cm^3^ CTV was 145.7, 116.98, and 139.23 cm^3^ for VMAT, IMPT_3%/3 mm_, and IMPT_3%/5 mm_, respectively.

The size of this dose cloud can be correlated to the CI of the different modalities. With greater conformality, IMPT treatment plans can reduce dose to nearby OARs; but the tighter dose profile leaves the plans more susceptible to variable CTV dosage when uncertainties are factored in. Both Figs. 1 and 2 show the appreciable differences in conformity for nominal and uncertainty plans. Robustly optimizing IMPT to 3 mm geometric uncertainties creates dose clouds that were always smaller than those of VMAT but created certain uncertainty scenarios in which the prescription dose cloud was smaller than the intended target volume. Alternatively, robustly optimizing IMPT to 5 mm geometric uncertainties did not necessarily (15.3%) result in nominal dose clouds small than that of VMAT but did result in these plans having CIs that were greater than unity in all uncertainty scenarios. Regardless of the deviations in the HI and CI under uncertainty situations, CTV coverage for both VMAT and IMPT maintained clinical acceptability. Thus, minimization of OAR dose should be the primary factor dictating treatment effectiveness between treatment modalities.

As anticipated, when uncertainties were minimized, IMPT plans become more favorable. If clinical workflows can maintain 3 mm uncertainties in the geometric reproducibility of a patient, IMPT plans were shown to be dosimetrically preferential to VMAT plans as they reduced irradiated volumes of both the anorectum and bladder at all investigated dose levels. This is true both for the nominal plans and for uncertainty plans.

If geometric reproducibility can only be maintained at 5 mm, IMPT was still shown to be preferable for minimizing irradiated volumes of the bladder and minimizing the volume of the anorectum that would receive intermediate to low doses, dose-volume levels that have been shown to be more predictive of rectal morbidity than smaller higher-dose regions [25]. At elevated dose levels, there was no statistical difference in the irradiated anorectum volumes for VMAT and IMPT plans with 5 mm uncertainties. These results indicate if geometric uncertainties can be kept below 5 mm there is potential for bladder and anorectum toxicities to be reduced with IMPT compared to VMAT, however clinical outcomes would be needed to validate this potential.

The areas of elevated anorectum dose in the IMPT treatment plans were exclusively from spots that had to be placed near the posterior edge of the CTV to achieve prescription coverage at the posterior border in both nominal and uncertainty scenarios. These spot placements pushed high dose into the anterior aspects of the anorectum. Although not used for patients in this study, the clinical usage of perirectal spacers could create greater separation between this posterior boundary and the anterior aspects of the anorectum and could provide significant high dose/small volume sparing of the anorectum for IMPT plans. Usage of these spacers would also allow for higher VMAT doses to be placed near the posterior boundary of the prostate without escalating dose to the anorectum.

There were several DV metrics whose mean values fluctuated minimally between nominal and uncertainty scenarios (less than 10% difference for all DV metrics except for V_8000cGy_ of anorectum for all plans, V_6500cGy_ for IMPT_3%/5 mm_ and VMAT_5mm_, and V_8000cGy_ of the bladder for VMAT_5mm_). This indicates that if geometric uncertainties are propagated in a truly random nature, then the nominal value of these DV metrics may be representative of the cumulative DV metrics from the summation of true day-to-day uncertainties. With this said, it is important to note that the probability of IMPT WCS is heavily right-skewed (left-modal) for OAR metrics (Figs. 3, 4). That is, although most uncertainty scenarios produce DV metrics that are similar to one another, there are a few WCS that exhibit much higher DV statistics. If the perturbations leading to the skewed nature of the uncertainty distribution presented themselves systematically, the cumulative DV metric for IMPT treatments over an entire course of radiation therapy could be much greater than the nominal metric. Further analysis could be warranted to investigate if the same perturbation type, perhaps a vertical shift, is causing the skewedness of the uncertainty distributions. This knowledge could aide in determining direction specific IMPT tolerances that would be allowed for in patient setup. Even though IMPT did have greater skewing than VMAT plans, the absolute values of the most extreme WCS were generally lower for IMPT plans.

All IMPT optimization in this study was carried out using MFO, which may be partially culpable for the increased HI of IMPT plans under uncertainty conditions. This study was not designed to compare between SFO and MFO techniques to determine if this increase in HI is a result of the intrinsic deposition properties of protons in IMPT or of the specific optimization routine used. Further study would need to be carried out to determine if MFO does increase dose heterogeneity compared to SFO for simple geometric target volumes such as the prostate, and importantly, if the increased heterogeneity corresponds with a decrease in OAR doses under uncertainties.

The uncertainties used it this study were selected to best represent widely used clinical standards. Geometric uncertainties of non-mobile tumors of 3 mm and 5 mm are commonly used, and these also correspond to the 3 mm and 5 mm PTV expansions in VMAT planning, which are used at our institution. A default range uncertainty of 3% is used at our institution for all treatment beams not traversing the lung and has previously been shown to be suitable for up 95% of prostate treatment plans [26]. With this said, it is not uncommon for institutions to use greater values for range uncertainties. However, as range uncertainty is only manifested parallel to the beam angle and the IMPT treatments plans only consisted of lateral beams, an increased range uncertainty would have minimal impact on lateral dose penumbra that is primarily responsible for OAR doses, although it could affect CTV coverage.

## Conclusion

This study demonstrates RO-IMPT plans for localized prostate cancer have clear dosimetric improvements of OAR sparing over that of VMAT plans in nominal and uncertainty situations up to 3 mm. Accounting for 5 mm of uncertainty, OAR sparing is generally better for IMPT planning, but statistical differences cannot be found at certain high dose volumes. In a clinical setting IMPT may be warranted for localized prostate cancer if geometric uncertainties can be kept to less than 5 mm.

## Data Availability

All references are cited in the manuscript. There is no additional data and material involved.
